# Locally advanced pancreatic carcinoma with jaundice: the benefit of a sequential treatment with stenting followed by CT-guided ^125^I seeds implantation

**DOI:** 10.1007/s00330-021-07764-6

**Published:** 2021-02-25

**Authors:** Chao Chen, Wei Wang, Wujie Wang, Yongzheng Wang, Zhe Yu, Yuliang Li

**Affiliations:** 1 Department of Interventional Medicine, The Second Hospital, Cheeloo College of Medicine, Shandong University, 247 Bei yuan road, Jinan, 250033 China; 2 Interventional Oncology Institute of Shandong University, Jinan, China

**Keywords:** Pancreatic carcinoma, Obstructive jaundice, Stents, Brachytherapy

## Abstract

**Objectives:**

To evaluate the role of sequential therapy with percutaneous biliary stenting and CT-guided iodine-125 seed implantation for locally advanced pancreatic carcinoma with concomitant obstructive jaundice.

**Methods:**

Between January 2016 and December 2018, 42 patients diagnosed with locally advanced pancreatic carcinoma with concomitant obstructive jaundice were enrolled retrospectively. All patients received biliary stenting via percutaneous transhepatic biliary drainage (PTBD) to alleviate obstructive jaundice. Thereafter, twenty-two patients underwent CT-guided iodine-125 seed implantation (treatment group), and 20 did not (control group). The prescribed dose in the treatment group was 110–130 Gy. The clinical data, duration of biliary stent patency, and overall survival (OS) were evaluated.

**Results:**

Overall, the total bilirubin level decreased from 275.89 ± 115.44 to 43.08 ± 43.35 μmol/L (*p* < 0.001) 1 month after percutaneous biliary stenting. In the treatment group, the postoperative median dose covering 90% of the target volume was 129.71 Gy. Compared with the control group, the treatment group had a long mean duration of biliary stent patency and median OS (11.42 vs. 8.57 months, *p* < 0.01; 11.67 vs. 9.40 months, *p* < 0.01, respectively). The overall positive response rates 6 months post-treatment in the treatment and control groups were 72.7% (16/22) and 30% (6/20), respectively. Adverse events of more than grade 3 were not observed during the follow-up.

**Conclusion:**

Sequential therapy with percutaneous biliary stenting and CT-guided iodine-125 seed implantation is an effective and safe treatment alternative for locally advanced pancreatic carcinoma with concomitant obstructive jaundice, which is worthy of clinical application.

**Key Points:**

*• Obstructive jaundice was alleviated after biliary stent placement in all patients, and the total bilirubin level decreased.*

*• The overall positive response rates at 6 months post-treatment were higher in the treatment group than in the control group, and adverse events of more than grade 3 were not observed during the follow-up period.*

*• Sequential therapy with percutaneous biliary stenting and CT-guided iodine-125 seed implantation can prolong biliary stent patency and improve survival.*

## Introduction

Pancreatic carcinoma is one of the most malignant tumors, because of its delayed diagnosis, aggressive tumor biology, and poor survival rate [1]. The symptoms of pancreatic carcinoma are usually non-specific; however, most patients simultaneously develop obstructive jaundice during initial diagnosis [2]. Only 20% of patients have the chance to undergo surgery [3], as most of them are in the terminal stage. The 5-year overall survival (OS) rate of patients with locally advanced pancreatic carcinoma is less than 5% [4].

Obstructive jaundice is one of the common symptoms of pancreatic carcinoma, especially in locally advanced pancreatic carcinoma, which could lead to liver dysfunction and eventually failure [5]. As this symptom can delay tumor treatment and increase the death rate, biliary obstruction should be urgently alleviated for further treatment. Percutaneous transhepatic biliary drainage (PTBD) has been performed to treat obstructive jaundice [6]. Self-expandable metallic stents are more comfortable than PTBD for patients, as they can avoid catheter dislodgement, cholangitis, and bile leakage [7]. The stents can reduce the serum bilirubin levels and improve patients’ quality of life and prognosis with malignant obstructive jaundice [8].

Combined gemcitabine chemotherapy is the most common therapy for locally advanced pancreatic carcinoma; however, its treatment outcomes have not been satisfactory [9]. The tumor can advance locally, leading to the re-obstruction of the biliary stent and reducing patients’ quality of life [10].

Recently, the implantation of radioactive seeds has been effective for the local control of many malignant tumors. The iodine-125 seeds can continuously release low-dose radiation to kill the tumor cells with reduced damage to the surrounding tissues. Studies have demonstrated that CT-guided iodine-125 seed implantation could serve as an effective and safe treatment for pancreatic carcinoma [11–13]. However, the treatment for locally advanced pancreatic carcinoma with concomitant obstructive jaundice is a significant challenge. Therefore, this study was performed to investigate the role of sequential CT-guided iodine-125 seeds brachytherapy for patients with locally advanced pancreatic carcinoma after alleviation of obstructive jaundice via percutaneous biliary stenting.

## Materials and methods

### Patients and scheme of treatment

Between January 2016 and December 2018, patients with locally advanced pancreatic carcinoma accompanied by carcinoma-related biliary obstruction were selected for this retrospective study. Patients had adequate cardiac, hematologic, liver, and renal function, and Eastern Cooperative Oncology Group (ECOG) performance status ≤ 2. Exclusion criteria were patients with severe cardiopulmonary dysfunction, ECOG performance status > 2, renal or liver failure, advanced cachexia, and tumors with diameters ≥ 7 cm or diffuse tumors. All patients received PTBD and biliary stenting for the treatment of obstructive jaundice. After biliary obstruction was alleviated, the advantages and disadvantages of iodine-125 seed implantation were introduced to all participants, who subsequently decided whether to receive iodine-125 seed implantation.

Finally, 42 patients were included in this retrospective study. Twenty-two patients chose the combined iodine-125 seed implantation therapy and were regarded as the treatment group. In contrast, 20 patients treated with PTBD alone were classified as the control group. Patients in the treatment group whose statuses were reassessed after PTBD underwent iodine-125 seed implantation when no contraindications were observed. All patients were recommended the combined gemcitabine chemotherapy after the procedure.

Ethical approval was obtained from the Second Hospital of Shandong University Regional Ethics Committee and written informed consent was obtained from all patients prior to the procedure.

### PTBD and stent placement

**Fig. 1:**
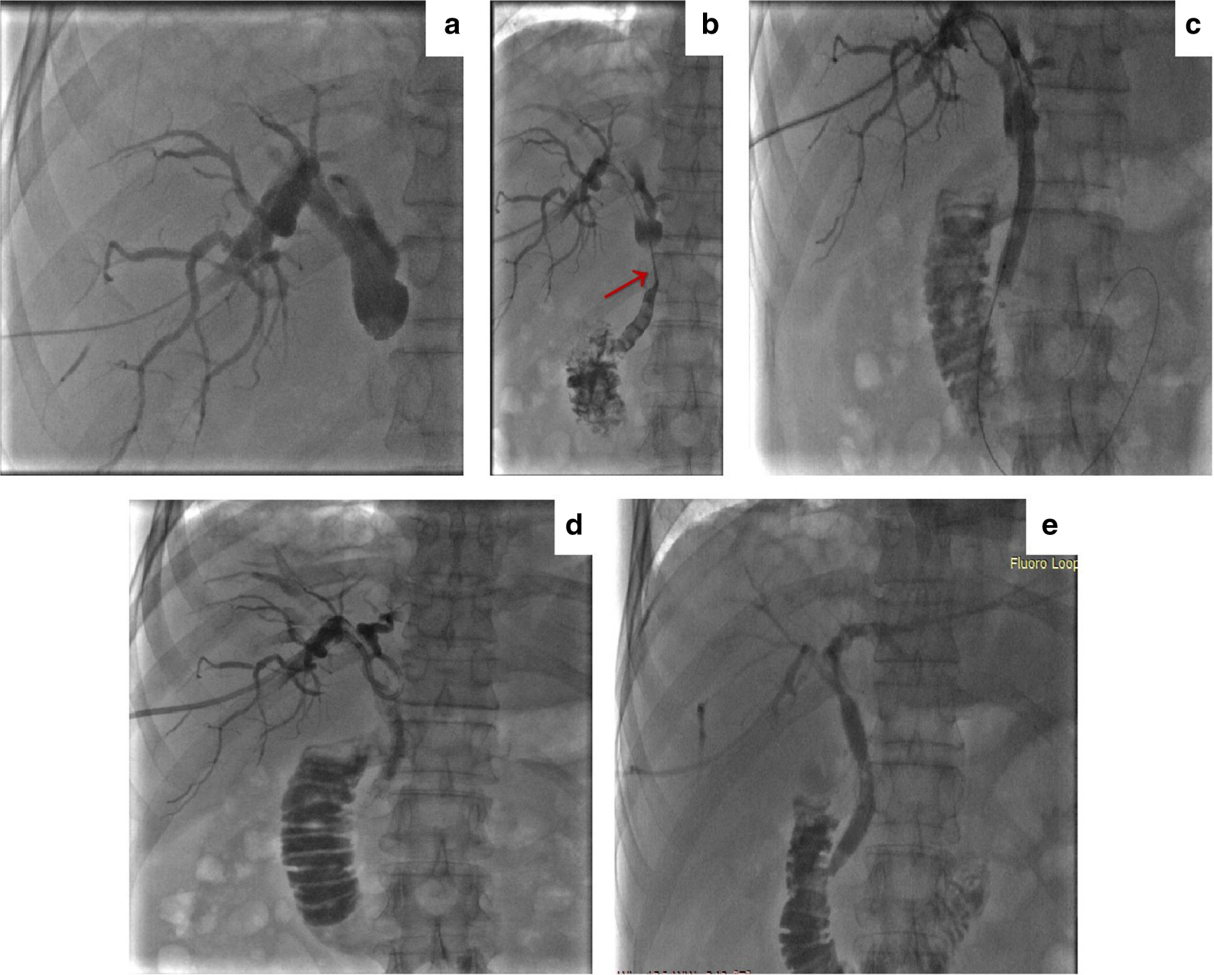
PTBD and stent placement procedure. **a** Cholangiography showing the obstruction of the common bile duct due to the pancreatic carcinoma. **b** Confirm the stenotic site and length of the bile duct (red arrow). **c** The stenotic site of bile duct was dilated with a balloon dilator catheter. **d** The placement of the biliary stent and 8.5 Fr drainage catheter. **e** Cholangiography showing that the stent was patent and well-expanded 1 week after the procedure

PTBD was performed under the guidance of ultrasonography or fluoroscopy. The patients were anesthetized using intravenous dexmedetomidine combined with a local anesthetic, lidocaine, before the procedure. Before biliary stent placement, the iodine contrast media was injected into the biliary tract through a 6-French (Fr) sheath and KMP catheter (Cook Medical Incorporated) to confirm the stenotic site and length of the bile duct (Fig. 1a and Fig. 1b). Subsequently, it was dilated with a balloon dilator catheter (Cook Medical Incorporated) (Fig. 1c). Self-expanding metallic stents (Zilver stents, Cook Medical Incorporated), including the proximal and distal adjacent portions, should be longer than the stricture to cover it completely. After stent placement, an 8.5-Fr drainage catheter (Cook Medical Incorporated) was retained in the bile duct through the puncture approach (Fig. 1d). About 1 week later, cholangiograms were obtained to evaluate the stent patency, and the drainage catheter was removed (Fig. 1e).

### CT-guided percutaneous iodine-125 seed implantation

**Fig. 2:**
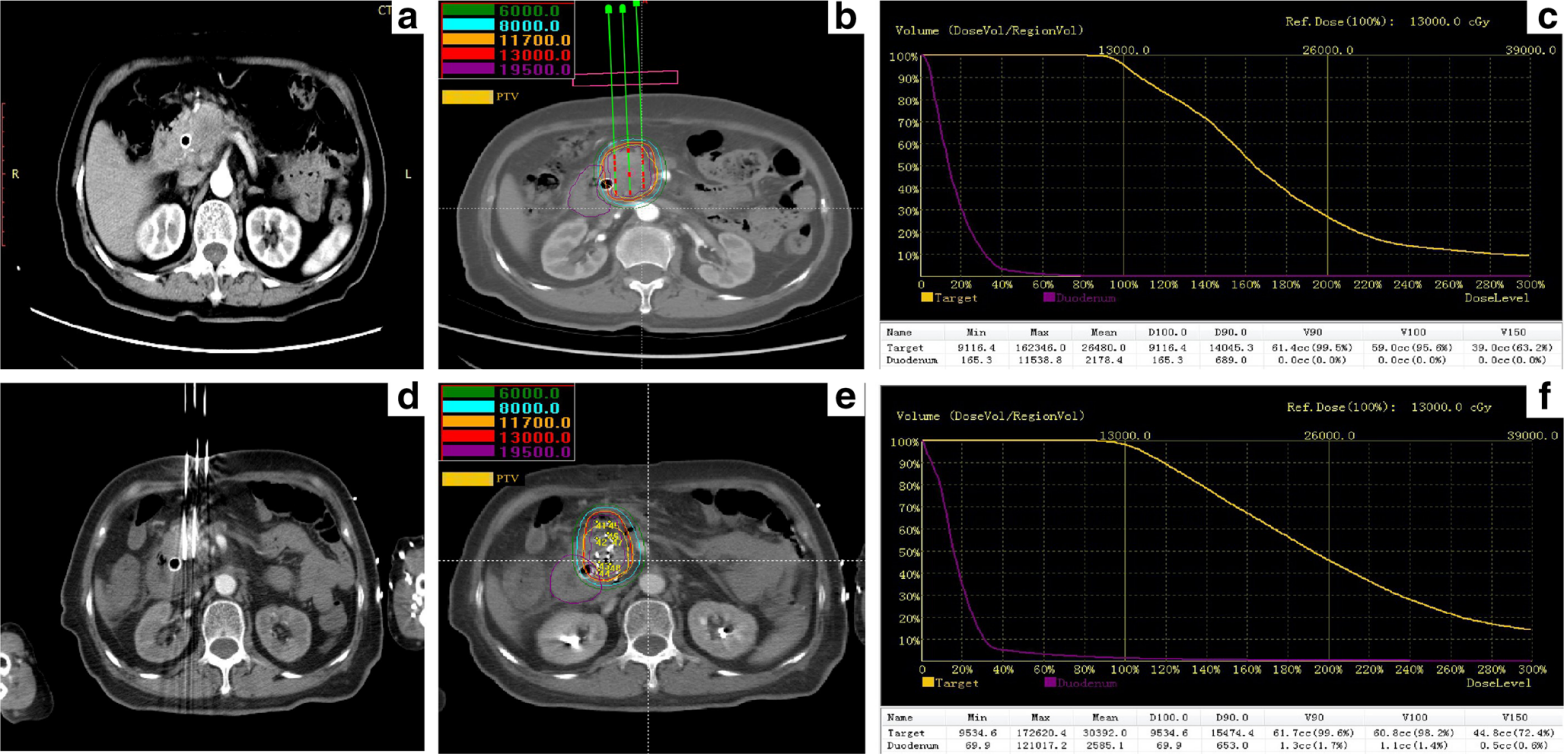
CT-guided percutaneous iodine-125 seed brachytherapy. **a** Preoperative computed tomography (CT) scan. **b**, **c** Treatment plan and the dose-volume histogram calculated before brachytherapy, the prescribed dose of this patient was 130 Gy. **d** Intraoperative puncture procedure under the CT-guided. **e**, **f** Coverage and the distribution of implanted seeds, dose distribution on treatment planning system and the dose-volume histogram calculated after brachytherapy. D90 was 154.74Gy and V100 was 98.2%. PTV, planning treatment volume; D90, the dose covering 90% of the target tumor volume; V100, the percentage of the target tumor volume covered by 100% prescribed dose

Routine upper abdominal contrast-enhanced CT (5-mm slice thickness) was performed preoperatively (Fig. 2a). Based on the CT findings, the radiation oncologist and surgeons outlined the gross tumor volume (GTV) and areas at risk for subclinical disease. The planning treatment volume (PTV) included the GTV and a 0.5–1.0 cm margin, and the prescribed dose was usually 110–130 Gy [14]. The distribution and dose of iodine-125 seeds were calculated using a computerized treatment planning system (TPS) (University of Beijing Aeronautics and Astronautics) (Fig. 2b). The TPS plan should consider the dose covering 90% of the target tumor volume (D90) ≥ the prescribed dose and the percentage of the target tumor volume covered by 100% of the prescribed dose (V100) > 95%. The exposure dose of the organ at risk (OAR) is also calculated to optimize the TPS plan (Fig. 2c).

Laxatives were administered, and a liquid diet was requested at night, 1 day preoperatively. Patients fasted the following morning and underwent preoperative enema and gastrointestinal decompression before brachytherapy. Broad spectrum antibiotics and analog of somatostatin were administered intravenously before the procedure.

Patients received intravenous anesthesia with dexmedetomidine and local anesthesia with lidocaine. According to the TPS plan, the needles (18 gauge; length, 150–200 mm; Hakko Medical Co. Ltd.) were implanted into the tumor according to the calculated depth and angle of direction (Fig. 2d). Subsequently, iodine-125 seeds (half-life: 59.4 days, penetration of 17 mm; Jaco Pharmaceuticals Co. Ltd.) were loaded and released. The entire procedure was performed under CT guidance. Finally, CT was performed again to assess the coverage and distribution of the implanted seeds and exclude any procedure-related complications, such as hemorrhage and migration of seeds (Fig. 2e and Fig. 2f).

Patients were requested to fast for about 72 h after the procedure. An analog of somatostatin and a proton pump inhibitor were administered intravenously, usually for 2–3 days or longer. Analgesics were administered to treat severe pain.

### Follow-up

Patients’ symptoms were evaluated before the follow-up. Postoperative follow-up was performed every 2 months thereafter. During each visit, blood laboratory examinations, contrast-enhanced CT or magnetic resonance imaging of the upper abdomen, and chest CT were conducted. Treatment efficacy was evaluated based on the duration of biliary stent patency and patients’ survival. The duration of stent patency was defined as the time from stent implantation to recurrence of obstruction or death from any cause. Local tumor responses were assessed using the Response Evaluation Criteria in Solid Tumors, version 1.1 [15]. The overall positive response was calculated as [complete response + partial response]/total number of patients ×100%.

### Statistical analysis

Continuous variables were expressed as mean ± standard deviation and were analyzed by the independent sample *t*-test. The paired-sample *t*-test was used to analyze continuous variables before and after treatment in the same group. The chi-square test was used to analyze the differences between ranked variables. Kaplan-Meier method was used to analyze survival and the duration of biliary stent patency, and a log-rank test was used to compare the significance of the differences between the groups. Univariate and multivariate Cox regression analyses were used to evaluate the relationship between different treatments and patients’ survival. Data were analyzed using SPSS, version 24.0 (IBM-SPSS), with all *p* values < 0.05 considered statistically significant for all tests.

## Results

Forty-two patients were included in this study, and patients’ data and characteristics are displayed in Table 1. Differences in age, sex, tumor location and stage, and levels of total bilirubin (TBIL), alanine transaminase (ALT), and hemoglobin (Hb) before the treatment were not statistically significant between the two groups. The diameter of the biliary stent ranged from 8 to 10 mm, and the average length was 6.4 cm (range: 6–8 cm).

**Table 1:** The characteristics of the patients

Characteristics	Treatment group (*n* = 22)	Control group (*n* = 20)	*p* value
Age (mean ± SD), year	63.41 ± 11.73	69.25 ± 12.75	0.13
Sex (male:female)	12:10	12:8	0.72
Location of tumors			0.95
Head	14	14	
Neck	5	4	
Body	2	1	
Whole pancreas	1	1	
Tumor stage			0.67
Stage II B	3	2	
Stage III	15	16	
Stage IV	4	2	
Total bilirubin (umol/L)	272.69 ± 124.60	279.41 ± 107.58	0.85
Alanine transaminase (ALT) (u/L)	141.83 ± 102.83	174.70 ± 134.55	0.38
Hemoglobin (g/L)	118.59 ± 15.89	112.05 ± 20.04	0.25

The treatments were successfully completed in all patients. Obstructive jaundice was evidently alleviated in all 42 patients after biliary stent placement. The TBIL level decreased from 275.89 ± 115.44 to 43.08 ± 43.35 μmol/L (*p* < 0.001) in all patients at 1 month after PTBD, from 272.69 ± 124.60 to 35.26±22.28 μmol/L (*p* < 0.001) in the treatment group, and from 279.41 ± 107.58 to 51.68 ± 58.13 μmol/L (*p* < 0.001) in the control group; no significant difference in the TBIL level was observed between the two groups. Liver function improved after treatment in both groups, with the ALT level decreasing from 157.48±118.67 to 103.21 ± 89.50 μ/L (*p* < 0.01) at 3 days after the primary treatment (Table 2).

**Table 2:** Comparison of laboratory examination in the two groups before and after treatment

Group	Percutaneous biliary stenting									Iodine-125 seed implantation		
	Total bilirubin (TBIL) (umol/L)			Alanine transaminase (ALT) (u/L)			Hemoglobin (Hb) (g/L)			Hemoglobin (Hb) (g/L)		
	Before	1 month after	*p* value	Before	3 days after	*p* value	Before	3 days after	*p* value	Before	3 days after	*p* value
Treatment group (*n* = 22)	272.69 ± 124.60	35.26 ± 22.28	< 0.001	141.83 ± 102.83	87.68 ± 66.41	< 0.01	118.59 ± 15.89	112.54 ± 17.13	0.004	116.23 ± 16.60	111.32 ± 16.62	0.065
Control group (*n* = 20)	279.41 ± 107.58	51.68 ± 58.13	< 0.001	174.70 ± 134.55	120.28 ± 108.75	< 0.01	112.05 ± 20.04	106.35 ± 16.43	0.027	*NA*	*NA*	*NA*
Total (*n* = 42)	275.89 ± 115.44	43.08 ± 43.35	< 0.001	157.48 ± 118.67	103.21 ± 89.50	< 0.01	115.48 ± 18.07	109.59 ± 16.89	< 0.01	*NA*	*NA*	*NA*

*NA*, not available

In the treatment group, the number of seeds implanted ranged from 24 to 90, with a median of 59 pellets. The specific activity of the seeds ranged from 0.48 to 0.62 mCi. The postoperative median dose of D90 was 129.71 Gy (95% confidence interval (CI): 126.17–133.24 Gy).

**Fig. 3:**
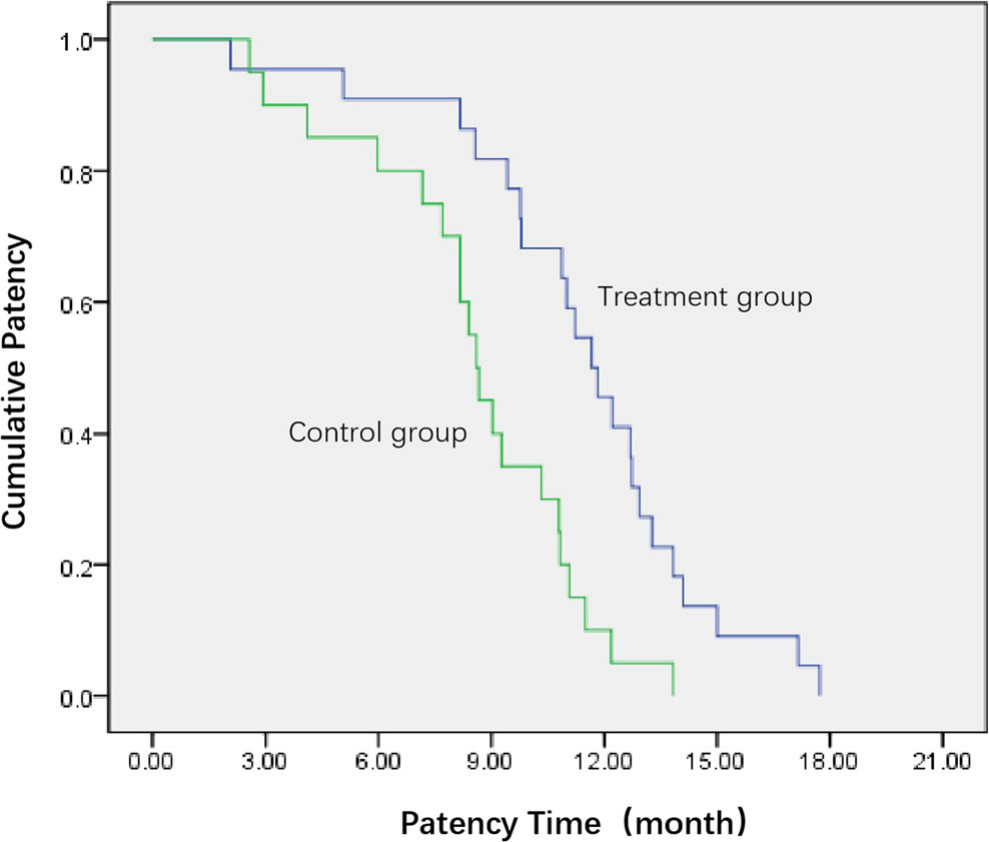
Stent patency time analysis. Mean duration of biliary stent patency was 11.42 months in the treatment, vs. 8.57 months in the control groups (*p* < 0.01, log-rank test)

The median duration of follow-up was 10.55 months (range, 2.07–21.17 months). The mean and median durations of biliary stent patency were 11.42 months (95% CI: 9.93–12.90 months) and 11.67 months (95% CI: 10.26–13.08 months) in the treatment group, and 8.57 months (95% CI: 7.26–9.87 months) and 8.60 months (95% CI: 8.01–9.19 months) in the control groups, respectively (Table 3); a significant difference was observed (*p* < 0.01) (Fig. 3). Seventeen and two patients in the control and treatment groups, respectively, developed obstructive jaundice again during the follow-up, and a significant difference was observed (*p* < 0.01).

**Table 3:** Comparison of stent patency and survival between two groups

	Duration of stent patency (months)					Overall survival (months)					
Kaplan-Meier analysis	Mean		Median		*p* value	Mean		Median		*p* value	12 months survival rate (%)
	Estimates	95% CI	Estimates	95% CI	0.001	Estimates	95% CI	Estimates	95% CI	0.004	
Treatment group	11.42	9.93–12.90	11.67	10.26–13.08		11.67	10.94–13.31	11.67	10.52–12.81		45.45%
Control group	8.57	7.26–9.87	8.60	8.01–9.19		9.32	8.04–10.59	9.40	8.61–10.19		10%

*CI*, confidence interval

**Fig. 4:**
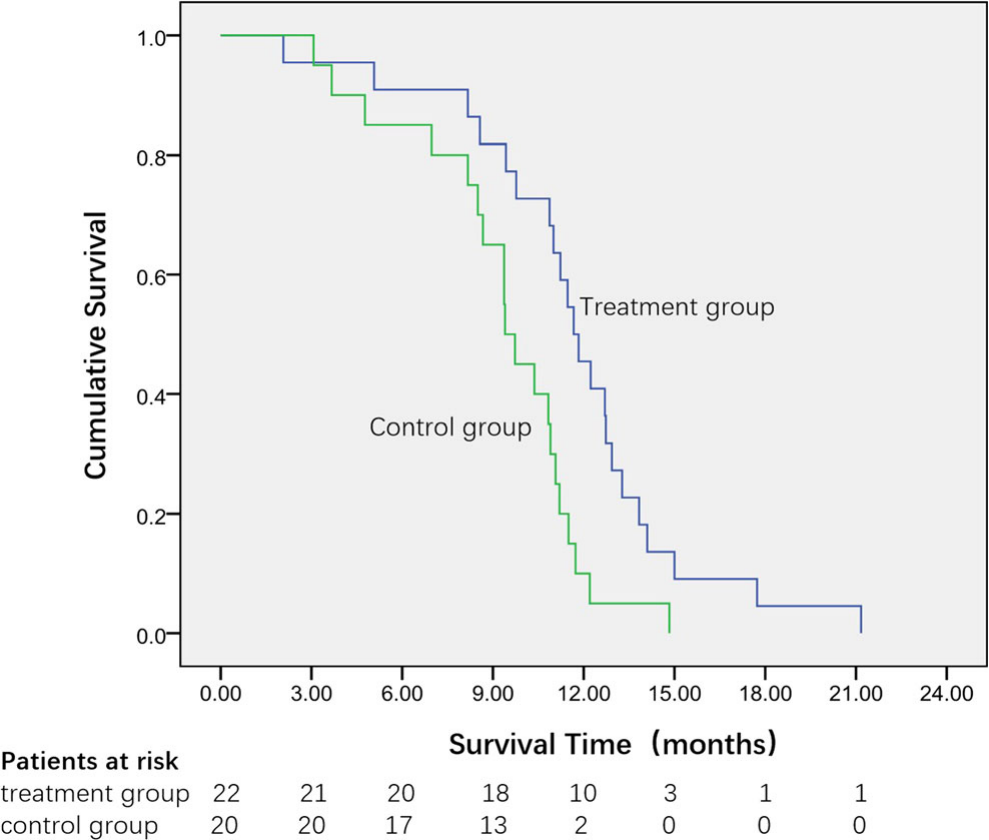
Survival curves of the two groups. Median overall survival was 11.67 months in the treatment group, vs. 9.40 months in the control group (*p* < 0.01, log-rank test)

The mean and median OS of the treatment group were 11.67 months (95% CI: 10.94–13.31 months) and 11.67 months (95% CI: 10.52–12.81 months), respectively. The mean and median OS of the control group were 9.32 months (95% CI: 8.04–10.59 months) and 9.40 months (95% CI: 8.61–10.19 months), respectively (Table 3). The difference in the OS between the groups was significant (*p* < 0.01), as shown by the Kaplan-Meier cumulative curves (Fig. 4), which illustrates that patients who underwent sequential therapy had better survival than those who did not. The similarity in survival persisted in univariate Cox regression analysis (Table 4). The multivariate Cox regression model showed that sequential therapy remained an independent prognostic factor for better survival (hazard ratio 0.281, 95% CI 0.134–0.598, *p* < 0.01). One-year survival rates were 45.45% (10/22) and 10% (2/20) in the treatment and control groups, respectively.

**Table 4:** Univariate and multivariate analysis of survival in patients who underwent different treatments

	HR	95% CI	*p* value
Univariate analysis			
Sequential therapy*	0.395	0.204–0.763	0.006
Percutaneous biliary stenting only	1		
Multivariate analysis^#^			
Sequential therapy*	0.281	0.134–0.589	0.001
Percutaneous biliary stenting only	1		

*Percutaneous biliary stenting combined with CT-guided iodine-125 seed implantation

^#^Cox regression model controlling for sex, age, location of tumors, and tumor stage

*HR*, hazard ratio; *CI*, confidence interval

**Fig. 5:**
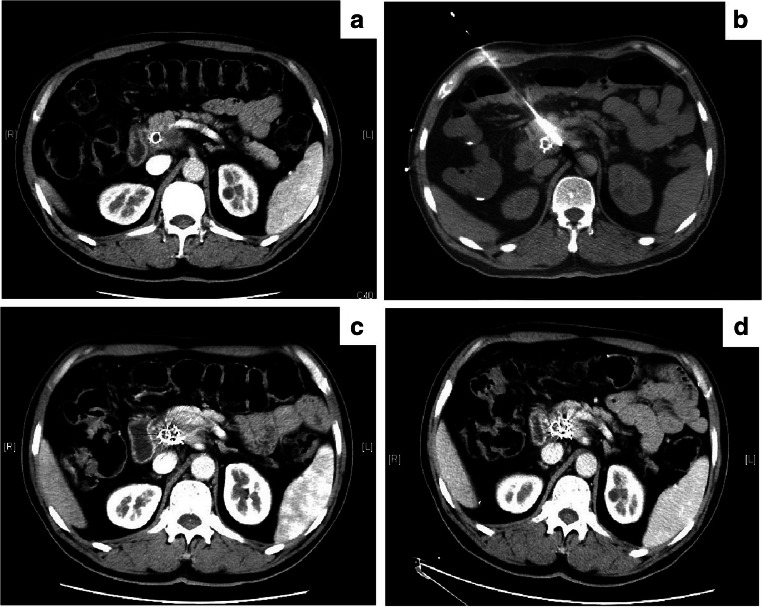
A 60-year-old male with pancreatic cancer at the neck, and the spleen artery was involved in. **a** Abdominal CT revealed the tumor before the treatment. **b** Intraoperative puncture procedure under the CT-guided. **c**, **d** The local lesion achieved complete response (CR) after 2 months (**c**) and 6 months (**d**) follow-up

**Fig. 6:**
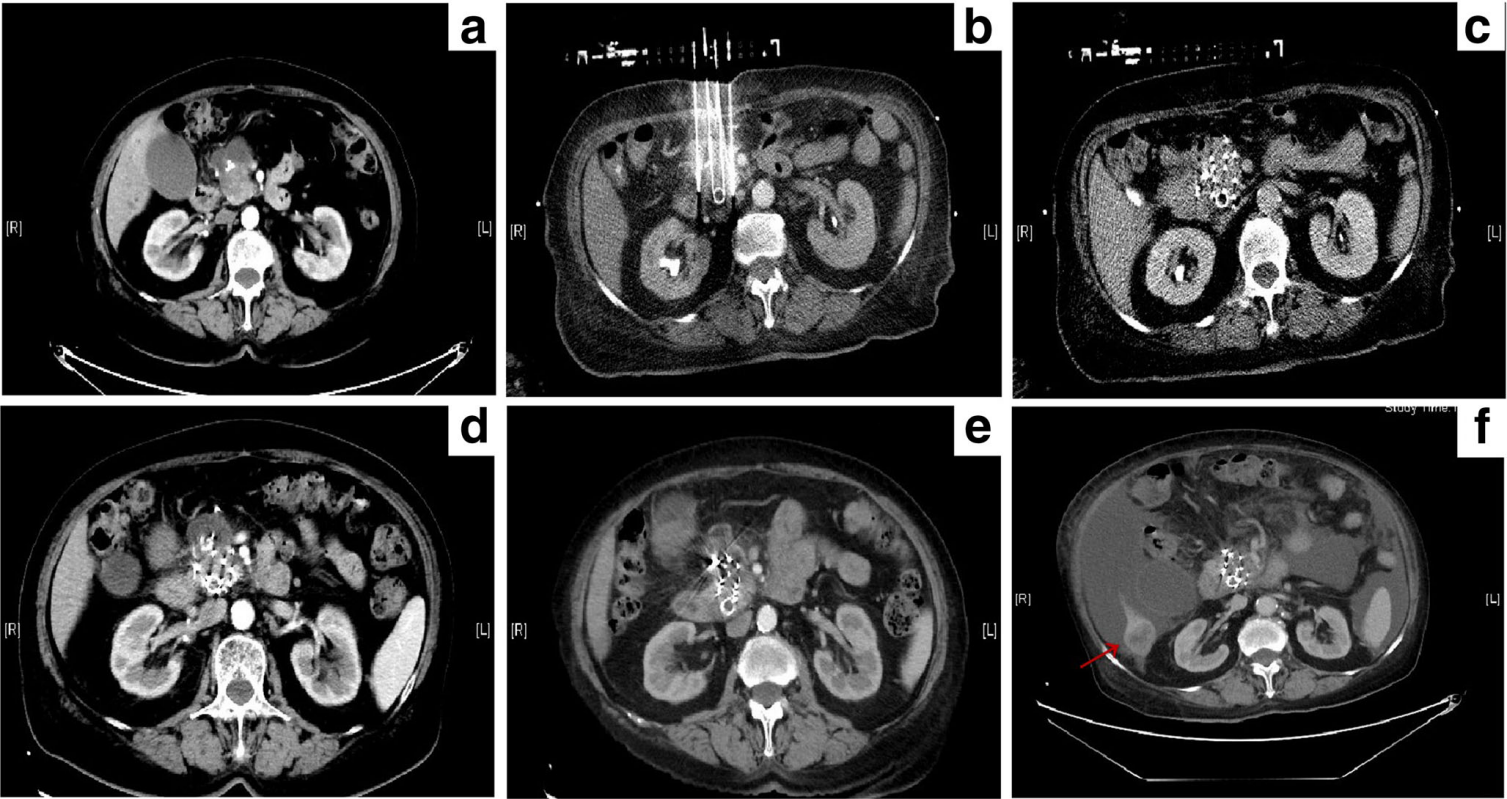
A 75-year-old female with pancreatic cancer at the junction of the body and pancreas head, and the superior mesenteric artery was involved in. **a** Abdominal CT revealed the tumor before the treatment. **b** Intraoperative puncture procedure under the CT-guided. **c** Coverage and the distribution of implanted seeds after brachytherapy. **d**, **e** The local lesion achieved complete response (CR) after 2 months (**d**) and 6 months (**e**) follow-up. **f** The patient has survived 17.73 months, and died of cachexia. Though there were liver metastasis (red arrow) and malignant ascites, the local lesion had been still under the control

Most patients in the treatment group had satisfactory local control of pancreatic carcinoma, which can be attributed to the iodine-125 seeds brachytherapy. Six months after the follow-up, the rates of complete response (CR), partial response (PR), stable disease (SD), and progressive disease (PD) were 27.3% (6/22), 45.4% (10/22), 18.2% (4/22), and 9.1% (2/22) in the treatment group and 0% (0/20), 30% (6/20), 45% (9/20), and 25% (5/20) in the control group, respectively. The rates of the overall positive response (CR+PR) in the treatment and control groups were 72.7% (16/22) and 30% (6/20), respectively. Two representative cases are shown in Fig. 5 and Fig. 6.

Complications of PTBD were transient abdominal pain (5/42), hemobilia (2/42), and cholangitis (4/42). Vomiting (3/42), low-grade fever (3/42), and abdominal distension (2/42) were similarly observed (Table 5). The Hb level decreased after PTBD (Table 2); however, most patients had no procedure-related symptoms, and no patient required blood transfusion. The abdominal pain and cholangitis were treated with conservative management, such as the administration of analgesics and antibiotics. Other minor complications were cured by symptomatic treatment.

**Table 5:** Procedure-related complications

Procedure Complication	Percutaneous biliary stenting (*n* = 42)	Iodine-125 seed implantation (*n* = 22)
Transient pain	5	3
Vomiting	3	4
Low-grade fever (< 38.0 °C)	3	3
Abdominal distension	2	6
Cholangitis	4	0
Pancreatitis	0	1
Hemobilia	2	0
Perforation	0	0
Seed migration	0	5
Pancreatic fistula	0	0
Intestinal fistula	0	0
Radiation duodenitis	0	0

The Hb level also decreased after iodine-125 seed implantation (Table 2); however, no significant difference was observed (*p* = 0.065). Seed migration into the liver occurred in 5 patients in the treatment group, and migration-related complications were not observed. In the perioperative period of brachytherapy, many patients developed symptoms, including puncture-related pain (3/42), vomiting (4/42), low-grade fever (3/42), and abdominal distension (6/42) (Table 5). All these problems were cured by symptomatic treatments, such as the administration of analgesics, antiemetics, mucosal protective agents, and gastrointestinal motility agents. Adverse events of more than grade 3 were not observed. Only 1 patient in the treatment group developed pancreatitis after brachytherapy, and she recovered well with the use of somatostatin. Serious complications such as pancreatic fistula, intestinal fistula, and radiation duodenitis were not observed during the follow-up period. In addition, treatment-related mortality was not noted.

## Discussion

Patients with locally advanced pancreatic carcinoma usually lose the chance to surgery and suffer from poor survival. About 70% of patients simultaneously develop biliary obstruction at the time of initial diagnosis with pancreatic cancer [2]. The presence of jaundice in pancreatic carcinoma is often associated with poor prognosis. PTBD can reduce jaundice and improve patients’ status; however, its efficiency is restricted by its catheter-related complications [16]. Stent implantation can prevent such problems and improve the patients’ quality of life [17]. Stent patency is an independent risk factor for patients’ survival [18]. Unfortunately, the duration of stent patency is limited due to tumor progression [19]. Experts have made efforts to guarantee the duration of stent patency. A covered metal stent was developed to overcome such problems. However, there is no significant difference in primary stent patency and stent dysfunction between covered self-expandable metal stents (CSEMSs) and uncovered self-expandable mental stents (UCSEMSs) [20]. Meanwhile, CSEMSs had a higher stent migration rate than UCSEMSs [21]. Studies [18, 22, 23] have also shown that compared with stent placement alone, placement of an irradiation stent could provide survival benefit and prolong stent patency in malignant obstructive jaundice. However, unlike biliary malignant tumors, obstructive jaundice caused by pancreatic carcinoma is often eccentric stenosis due to tumor oppression. Hence, in most cases, it is difficult to achieve the prescribed dose by relying on the radioactive seeds carried by biliary stent. Further, because of the distance of irradiation, it is difficult to effectively kill the tumor cells distal from the biliary tract. Previous studies suggested that CT-guided percutaneous implantation of iodine-125 seeds is relatively safe and effective for treating locally advanced pancreatic carcinoma, without additional complications [13]. However, a few studies have assessed the role of percutaneous biliary stenting and iodine-125 seed implantation for locally advanced pancreatic carcinoma accompanied with obstructive jaundice. In this study, we conducted a sequential therapy for these patients and achieved a good therapeutic effect.

Self-expandable metallic stents provide symptomatic relief and prevent catheter-related complications. Firstly, we implanted the stent at the stenotic site of the common bile duct due to pancreatic carcinoma to resolve the obstruction. Obstructive jaundice improved after stent implantation. Moreover, liver function similarly improved after the procedure. Although the Hb level decreased, blood transfusion was not necessary. Transient abdominal pain, cholangitis, and abdominal distension can be controlled by symptomatic treatment. Percutaneous biliary stenting is safe and effective in alleviating the obstructive jaundice, providing the opportunity for further treatment. Moreover, iodine-125 seed implantation was conducted as a sequential therapy, after the alleviation of obstructive jaundice. The Hb level also slightly decreased after iodine-125 seeds brachytherapy, but no significance and related symptoms were observed. No patient developed severe complications. Patients benefitted from adequate preoperative preparation and somatostatin application during the perioperative period, and only 1 patient experienced pancreatitis. Most complications were tolerable and controlled by conservative treatment. The treatment group had a significantly longer mean duration of biliary stent patency and median OS than the control group (11.42 vs. 8.57 months, *p* < 0.01; 11.67 vs. 9.40 months, *p* < 0.01, respectively). Furthermore, sequential therapy was an independent prognostic factor for better survival in multivariate analysis.

Iodine-125 seed irradiation could promote cell apoptosis and inhibit pancreatic cancer growth [24]. Meanwhile, combined treatment of iodine-125 seed and gemcitabine has a stronger anti-proliferation effect than either monotherapy (gemcitabine or iodine-125 seed alone) in PANC-1 cells [25]. In addition, this may be the reason why treatment with iodine-125 seed implantation resulted in better survival and higher local control rate in the treatment group.

Systemic chemotherapy is the standard treatment for locally advanced pancreatic carcinoma. Additionally, the median OS ranged from 6.7 to 12.0 months in different trials [9, 26–28] based on the combined gemcitabine chemotherapy. Wang et al reported that the median OS was 10.31 months for unresectable pancreatic cancer by CT-guided brachytherapy using iodine-125 seed implantation [29]. In our study, the median OS was 11.67 months in the treatment group. This sequential therapy could ensure a good survival in patients with locally advanced pancreatic carcinoma with concomitant obstructive jaundice.

Pancreatic carcinoma is not so sensitive to radiotherapy. It needs a high cumulative dose of radiotherapy to obtain a satisfactory curative effect [30]. However, the radiation dose rate is limited by the surrounding tissues. According to the National Comprehensive Cancer Network clinical practice guidelines for pancreatic carcinoma, the recommended dose generally includes 45–54 Gy in 1.8–2.0 Gy fractions [31]. The implanted iodine-125 seeds can generate a high dose (120–160 Gy) within the target tumors. The median D90 in our study was 129.71 Gy, which was high enough to kill the tumor cells. Although the local irradiation dose was high, the surrounding non-neoplastic tissues only received a very low dose because of the short irradiation distance of the radioactive seeds. Radiation duodenitis was not observed during the follow-up. Currently, endoscopic therapy is recommended for the alleviation of obstruction in pancreatic carcinoma [32]. However, we prefer to insert the biliary stent via PTBD, as it has better clinical safety in our experience. Iodine-125 implantation under CT guidance rather than endoscopic ultrasound and/or ultrasound guidance should be adopted initially, as it is less disturbed by intestinal gas and respiration. Importantly, the CT scan can guarantee a better follow-up of the preoperative TPS plan for puncture and seed implantation, ensure perioperative safety, and reduce the incidence of complications.

Nevertheless, this study has a few limitations. First, this was a retrospective study; further multicenter and randomized controlled trials are needed. In addition, the number of patients should be increased in future studies to increase the generalizability of the study findings. Lastly, the application of these techniques requires abundant clinical experience on the researchers’ part.

In conclusion, we revealed an effective and safe sequential therapy for patients with locally advanced pancreatic carcinoma with concomitant obstructive jaundice. Percutaneous biliary stenting can relieve the jaundice and improve liver function. Thereafter, the use of iodine-125 seed implantation should be considered since it can not only prolong the biliary stent patency, but also improve survival.

## Abbreviations

ALT: Alanine transaminase
CI: Confidence interval
CR: Complete response
D90: The dose covering 90% of the target tumor volume
ECOG: Eastern Cooperative Oncology Group
Fr: French
GTV: Gross tumor volume
Hb: Hemoglobin
OAR: Organ at risk
OS: Overall survival
PTBD: Percutaneous transhepatic biliary drainage
PTV: Planning treatment volume
TBIL: Total bilirubin
TPS: Treatment planning system

## Declarations

### Guarantor

The scientific guarantor of this publication is Yuliang Li.

### Statistics and biometry

One of the authors has significant statistical expertise.

### Methodology

- Retrospective
- Observational
- Performed at one institution
